# An Inquiry-Based Lab Exercise Investigating Student Cortisol Responses to Giving a Graded In-Class Presentation

**DOI:** 10.59390/001c.157973

**Published:** 2026-04-23

**Authors:** Nicole M. Baran

**Affiliations:** 1 Department of Biology Davidson College

**Keywords:** neuroendocrinology, cortisol, stress, psychosocial stressors, lab activity, inquiry-based

## Abstract

Inquiry-based lab exercises have been shown to enhance student scientific skills and confidence, increase inclusivity for underrepresented populations, and improve persistence in science. Here I share the results and learning outcomes of an inquiry-based laboratory project investigating salivary cortisol responses to psychosocial stressors in an upper-level Comparative Neuroendocrinology course at a small liberal arts college. In this experiment, students collected saliva samples from themselves during a normal lab day (baseline) and then 10 minutes after giving a graded, in-person presentation on a scientific journal article. We used a short, student-generated survey to measure self-reported anxiety levels, feelings about public speaking, and current workload. In a subsequent lab exercise, students performed a simulated enzyme-linked immunosorbent assay (ELISA) which was designed to illustrate important concepts in ELISA design including the usage of the standard curve and the importance of proper controls. I then provided students with their own anonymized data and collaborated with them to carry out the statistical analysis of the class dataset. The students collectively found that the experience of giving a graded presentation significantly increased salivary cortisol concentrations compared to baseline and that the increase in cortisol is positively associated with students’ self-reported experience of stress in response to public speaking. The multiple lab exercises culminated in a summative assignment to design a clear scientific poster sharing the results. The project led to extremely high student motivation and provided students with hands-on experience using foundational neuroendocrinological methods—as both the researcher and the subject.

## INTRODUCTION

Inquiry-based lab exercises enhance student scientific skills and confidence, increase inclusivity for underrepresented groups, and improve persistence in science, technology, engineering and math (STEM) fields (Gormally et al., 2009). Incorporating these authentic science experiences in the classroom has the potential to lead to improvements in students’ science literacy and research skills, help students gain self-confidence in their scientific abilities, and increase student motivation. Indeed, authentic research experiences are one of several student-centered learning approaches recommended to improve learning in the sciences (American Association for the Advancement of Science (AAAS), 2010). There is a need for more hands-on lab experiences designed for neuroendocrinology, biological psychology, and behavioral neuroscience students that teach a range of applicable and interdisciplinary skills.

Here I share the results and learning outcomes of a course-based laboratory project investigating salivary cortisol responses to a psychosocial stressor—giving a graded, in-class presentation—in an upper-level neuroendocrinology course at a small liberal arts college. Students participated in the study both as researchers and participants and collaborated as a class to design the survey used in the experiment to measure their own stress, carried out a simulated enzyme-linked immunosorbent assay, and analyzed and interpreted class-generated data.

Prior to the start of the lab project, students first learned about the difficult-to-define concept of stress and the biology of the physiological stress response. In the course, we defined stress as a significant disturbance from homeostasis that provokes elevated arousal caused by something that is perceived as aversive and out of one’s control, following the model proposed by Kim and Diamond (2002). Stress activates the hypothalamic-pituitary-adrenal (HPA) axis (Nelson & Kriegsfeld, 2023). The primary function of the HPA axis is to release glucocorticoid hormones, which are steroid hormones released by the cortex of the adrenal glands. Glucocorticoids mediate many physiological functions via the mobilization of glucose resources (gluconeogenesis), reduction of inflammation, and myriad impacts on the brain.

A well-known function of the HPA axis is that it is the neuroendocrine link between a perceived, psychological stressor and the physiological response to stress (Dickerson & Kemeny, 2004). Psychosocial stressors are events or circumstances arising from social and interpersonal relationships that cause stress, which can range from the mundane (an exam or difficult conversation) to the catastrophic (trauma, abuse, or death of a loved one). These events have been shown to cause reliable and measurable increases in the hormone cortisol (CORT), the primary glucocorticoid in humans.

One of the most reliable methods that is used to elicit an increase in cortisol is the Trier Social Stress (TSS) test (Kirschbaum et al., 1993). In its original formulation, the TSS test involves the preparation of a presentation in the form of a job interview, giving the presentation, and then performing mental arithmetic—all in front of a panel of judges. The TSS test contains elements of both social-evaluative threat and uncontrollability, which has been shown to elicit large cortisol responses (Dickerson & Kemeny, 2004).

Cortisol can be non-invasively measured in saliva and this has become an extremely common assay used in human-subjects research. A search on PubMed for the term “salivary cortisol” yielded 7,113 results demonstrating the widespread use of this technique. Thus, students through this project are engaged in an extremely common methodological technique in psychology, neuroscience, and neuroendocrinology. Although salivary cortisol labs have been implemented in undergraduate classrooms (Kalman & Grahn, 2004), increased biosafety concerns with the coronavirus pandemic have created additional barriers for students to collect and work with human saliva samples themselves. I thus discuss alternative approaches to safely carry out this lab and meet many of the same learning objectives, which includes modular components that can be adopted into courses in part or in whole.

## MATERIALS AND METHODS

### Institutional Context, Participants, & Course Structure

Davidson College is a private 4-year undergraduate small liberal arts college that, at the time of the study, was 62% white, 10% Hispanic, 6% black, 5% Asian, 5% multi-racial, 1% race/ethnicity unknown, and 10% international. Approximately 17% of students at Davidson College were Pell Grant eligible.

All 32 students in two sections of an upper-level comparative neuroendocrinology course participated in the study/lab exercise. The course met twice/week for a 75min lecture and discussion and once/week for a 160min lab. There were two lab sections of 16 students each, which took place in the afternoons between 1:40-4:20pm. In the course, 9/32 (28.1%) students identified as male and 23/32 (71.9%) identified as female.

The learning objectives associated with the lab portion of the course were as follows:

1. Discuss and apply various approaches used in laboratory and experimental settings to obtain information about the relationship between hormones and behavior.
2. Develop testable hypotheses.
3. Design and carry out experiments or studies using appropriate sampling methods.
4. Analyze and interpret data.
5. Effectively communicate in oral and written formats, provide constructive feedback, and collaborate with their peers.

### Ethics & Biosafety Review Process

Students provided informed consent during both lab sections to participate in the study, which included engaging in the lab exercise as part of the class, providing saliva samples, completing the student-designed surveys, and analysis of outcomes associated with the activity. I used the opportunity to teach students about informed consent and Institutional Review Board (IRB) processes and regulations. As the instructor, I emphasized that participation in the study aspect of the exercise was entirely voluntary, that results would be anonymized, and that the students’ participation in the study was in no way linked to their course grade. Note that students were told that they could participate in the activity without providing consent to participate in the study. The data were deidentified—only the students knew the unique identifier associated with their individual data. No demographic data was linked to anonymous student codes, as this data would have been identifying given the class size and demographics. The study was approved by and conducted in accordance with the guidelines from the IRB at Davidson College (IRB # 2024-002). Additionally, the students in the course themselves created a survey to be completed during the study (see below). These survey questions were approved by the IRB as a planned modification to the protocol just prior to the start of the study.

Additionally, all procedures were approved by Davidson College’s Biosafety Committee. The key elements of the biosafety procedures that were in place included 1) training for the students in how to collect and transport saliva samples (which are human body fluid samples) without exposure to others, 2) biosafety training for the instructor and all staff who handled the samples, and 3) the ELISAs were carried out in a space and following procedures that adhered to Biosafety Level 2 (BSL-2) guidelines.

### In-Classroom Preparatory Work

Prior to the start of the lab project, the students engaged in lectures and discussions about the HPA axis and stress as part of the in-classroom work. In addition, they were assigned to read the ‘Stress’ chapter (Chapter 11) of the course textbook (Nelson & Kriegsfeld, 2023) and listen to a Radiolab podcast episode (Abumrad, 2007). At the end of the series of lectures, as the instructor, I used class time to guide the students in the process of designing a survey that they would complete during the lab immediately after providing the sample (see Supplementary Materials).

Students in the course were interested in asking questions about overall workload, commitments outside of the classroom, hours of sleep per night, as well as self-reported measures about anxiety experienced with public speaking and overall stress levels. I also nudged the students to think about questions both associated with short-term increases in stress/anxiety from giving the presentation compared to a control day, which resulted in the inclusion of the six-item short-form of the Spielberger State-Trait Anxiety Inventory (STAI) (Marteau & Bekker, 1992; Spielberger et al., 1983).

### Lab 1: Baseline Sample

During the first lab, students first received training in collecting the saliva sample using cortisol salivettes (Sarstedt #51.1534.500). They were additionally provided information about both the biosafety and IRB review process and provided informed consent to participate in the study. Each student received an instruction packet with detailed information about how to collect their sample (see Supplemental Materials), as well as a random 3-digit code. They were instructed that this code was unique to them and that they should keep it private to themselves to ensure that their and other students’ data would remain anonymized.

During the lab, students were assigned the task of working in randomly-assigned pairs to select a paper on ‘psychosocial stressors and salivary cortisol’ and then to read the paper and work with their partner to put together a 5–7-minute presentation on the paper that they would give during the lab period the following week. Students found papers on a wide range of different kinds of psychosocial stressors that are interesting to them, ranging from athletic performance to parenting stress, trauma, etc. Prior to this point in the course, the students have already engaged with the primary literature in the field and have completed an in-depth analysis of a primary journal article and they have received a list of high-quality journals in the field. However, I required that the students received verbal ‘approval’ of their selected paper from me before proceeding with putting together their presentation. I emphasized that the presentation would be graded, although the presentation accounted for a very small proportion of their final grade (less than 1% total).

The relatively calm, low-stakes activity during the first-week lab was designed to serve as the baseline (i.e. control) condition. During the lab, the pairs of students were randomly assigned an order in which they would provide their saliva sample. Salivary cortisol peaks about 20-30 minutes following the onset of a stressor (Dickerson & Kemeny, 2004; Smyth et al., 2013). Additionally, cortisol has a strong diurnal rhythm, peaking in the morning shortly after waking and then declining throughout the day (Pruessner et al., 1997).

In order to account for these factors, sample collection began ~30 minutes into the lab. Students would leave the lab in assigned pairs to each provide their individual sample in 10-minute intervals thereafter. This order and approximate timing were then replicated during the following lab when we collected the treatment samples following the presentations. Samples were collected in a nearby study room with adjacent sink that was closed for the dates/times of sample collection, but any location that is easily sanitized with access to a sink (such as a single-use bathroom or shower room) works well. Upon returning to the lab room, the students were asked to complete the student-designed survey via a Google Form.

### Lab 2: Treatment Sample

During the second lab, students gave their graded 5–7-minute presentations, collected their saliva samples, and completed the survey a second time. The samples occurred in the same order and, thus, the same approximate time as their control sample which was collected the week before. To mimic features of the original TSS test, I wore a lab coat (also associated with BSL-2 guidelines for handling saliva samples) and sat directly in the back of the room in the middle of the students’ eyeline during their presentations. I took notes on their presentation with a pen and clipboard using a short rubric, but I did not speak or provide comments except to encourage questions from the audience or note the time. Additionally, all students were required to ask at least two questions of their peers during the question-and-answer phase, so students also responded live to student questions as part of their graded presentations.

After completing their presentation, the student pairs were asked to set a timer for 10 minutes. When their timer went off, they went to collect their treatment saliva sample. This allowed us to capture the peak of cortisol following the presentation stressor. Upon returning to the classroom, the students were asked to complete the survey a second time.

### Lab 3: Simulated Enzyme-Linked Immunosorbent Assay (ELISA)

Ideally, the next step in an authentic research experience would be for students to directly use an enzyme-linked immunosorbent assay (ELISA) to measure cortisol in their saliva samples. However, this would present biosafety concerns, necessitating all students to obtain BSL-2 training. Instead, I developed a simulated ELISA using a modified protocol from a safe, commercially-available kit (Carolina Biologicals: 211248). The primary goals of the lab are for students to understand the 1) principles of competitive sandwich ELISAs (a common assay for many kinds of clinical testing), 2) the various plate controls when quantifying the amount of substance in sample, and 3) the standard curve. In this lab, I gave a short (~15min) lecture on competitive sandwich ELISAs and guided students through the process of completing a worksheet on how these ELISAs work.

Next, the students used pipettors and pipette tips to follow the directions for a simulated ELISA. This ELISA and the procedures were designed to mimic the principles of the same ELISA used to measure the salivary cortisol samples and includes the standard curve and the same controls. If the students follow the protocol directions carefully, the simulated ELISA results in a color change from turquoise to purple. In the simulated ELISA, the depth of the purple color is inversely proportional to the hypothetical concentration of cortisol in the sample and the differences in sample concentration can be seen with the naked eye. To encourage additional student motivation, the simulated ELISAs were run on a plate reader at 400 nm (violet light) and this was used to compute the standard curve and R^2^. Students who obtain the highest R^2^—which is correlated with pipetting accuracy and ability to follow the protocol—received a small prize. Preparation details and the student handout (including the student protocol) is available in the Supplementary Materials.

### Lab 4: Analyze Class Data

During the two weeks between Lab 2 and Lab 4, I assayed the student salivary cortisol concentration using an ELISA in a BSL-2 space. First, I used a commercially available salivary transferrin ELISA kit (Salimetrics #1-1302) to test for evidence of blood contamination which increases the hazards of the sample and interferes with the measurement of cortisol. Samples were run as single replicates and one sample was excluded for transferrin concentrations greater than 2mg/dL. Next, the cortisol concentrations were measured in the saliva samples using a commercial salivary cortisol ELISA kit (Salimetrics #1-3002; functional sensitivity 0.018µg/dL) following the manufacturer’s instructions. Samples were run in duplicate. Plates were run at 450 nm with a correction at 491 nm on a microplate reader. One sample was excluded for a coefficient of variation (%CV) greater than 20%. Additionally, there were two students that did not provide a saliva sample on one of the two sample days due to an absence or sickness. This led to a final sample size of 28. The mean intra-assay coefficient of variation (including all samples) was 2.8%; the inter-assay variation was 15.8% (average of the high and low cortisol control samples on the two plates).

During the class, I provided the students with the raw data from the plate reader, processed data where individual sample concentrations have been computed using the standard curve, as well as the anonymized survey results. Students were guided through the quality control steps above and we discussed the importance of running samples in duplicate. From this, the students built an analyzable dataset in Excel. From this, they were required to carry out two analyses: 1) a paired t-test testing the primary hypothesis of the lab (that salivary cortisol increases following the presentation compared to the control day) and 2) a linear regression testing whether salivary cortisol concentrations (or differences in cortisol concentrations from treatment to baseline) are linearly associated with one of the many numerical variables that we collected in the survey. Statistics was not a prerequisite for this class, though some students had more expertise than others. Both of these analyses can be completed in Excel or students may use other software if they are more familiar with these. The goal of the lab is for the students to successfully carry out these analyses before leaving lab, which allows them to complete the final project associated with the lab.

The final assessment for this lab was to present their results on a scientific poster, which included Introduction, Methods, Results, and Discussion components. However, I have in other semesters required students write and turn in a 2-page lab report instead. I prefer the lab report format in absence of an accompanying poster presentation.

## RESULTS

**Figure 1.**
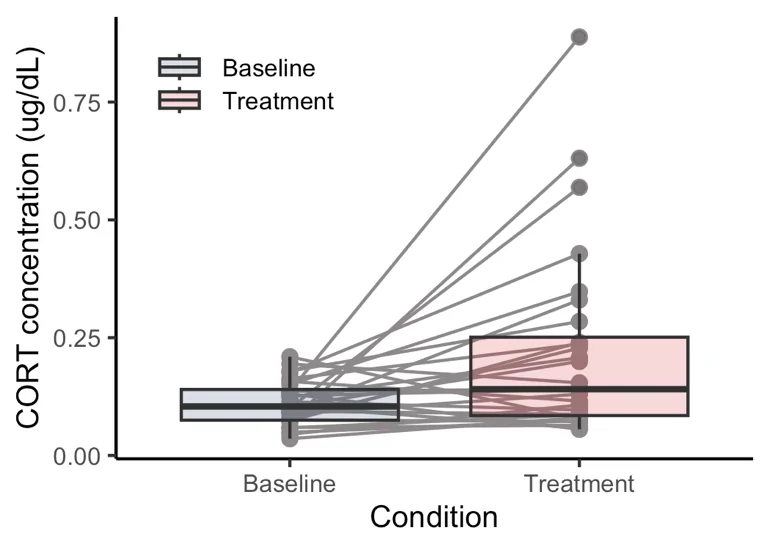
Giving an in-class presentation increases salivary cortisol concentrations in college students. Boxplot of salivary cortisol concentration in μg/dL from baseline condition (on normal lab day) and the treatment condition (10min following a graded in-class presentation to peers summarizing a scientific paper). Lines connect data points from the same individual.

Consistent with predictions, I (and the students!) found that salivary cortisol significantly increased in treatment samples taken after their graded presentation compared to the control samples taken the week before (Paired-sample t-test: *t*(27) = -2.8701, *p* = 0.0079, Fig. 1). Of the 28 students for whom we had complete data, 20 students showed a measurable increase in cortisol in the treatment condition.

**Figure 2.**
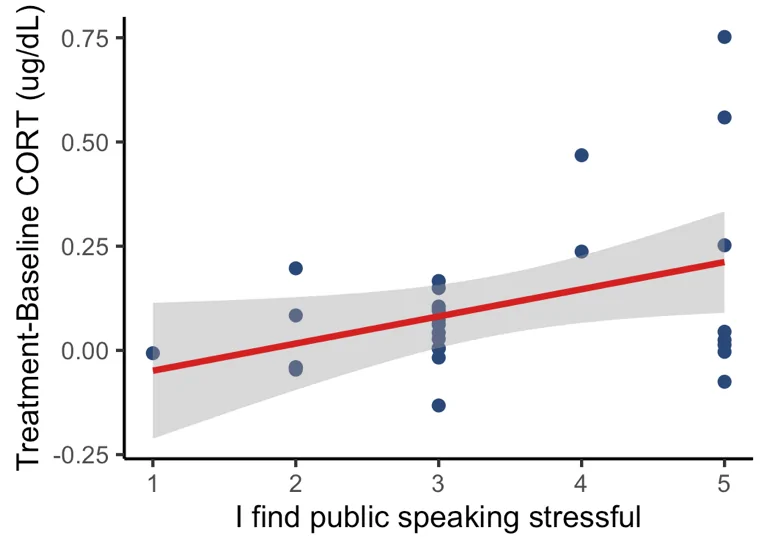
Salivary cortisol increase is associated with increased in self-reported stress with public speaking. Scatterplot of relationship between the change in cortisol (treatment-baseline cortisol in µg/dL) and the subjects’ response following baseline sample to the statement “I find public speaking stressful”, where 1=Strongly Disagree and 5=Strongly Agree. Red line shows the line of best fit. Shading represents the pointwise 95% confidence interval.

In addition, we found that there was a significant positive association between the students’ rating of the extent to which they found public speaking stressful and their difference in cortisol concentrations between treatment versus baseline (Linear regression: *F*(1,27) = 4.800, *p* = 0.0376, Fig. 2). The more highly the student rated the statement, ‘I find public speaking stressful’, the greater their increase in cortisol following the presentation.

Although the class investigated several possible numerical variables, no other variables reached statistical significance in this sample. There was, however, a non-significant trend for the change in the average state-trait anxiety inventory to be associated with the change in cortisol concentration from treatment compared to baseline (Linear regression: *F*(1,21) = 2.999, *p* = 0.098). I did not correct for multiple testing here, but it is worth noting the sample size was somewhat reduced due to some students not completing the survey both times.

### Course Evaluations and Student Reflections

In the final anonymous course evaluations for the semester, I counted 12 spontaneous positive mentions of the labs for the course (the course evaluation questionnaire did not ask specific questions about labs at all) and there were five positive spontaneous mentions of this cortisol lab, in particular. “I really liked the CORT lab. I thought that was really interesting and fun,” wrote one student. Another student wrote, “The labs were great, and I really enjoyed how they integrated with what we were reading and learning about in lecture.” Another student similarly commented on the connections between the lab and classroom portions of the course, “Lab activities were well-tailored to what we were learning in the course.” Another wrote of the lab’s connections to their real lives, “The creative labs and assignments helped connect concepts to real-world issues in an engaging way.”

In addition, at the end of the course, I asked students to anonymously write short letters to future students in the course. In contrast to course evaluations, this activity often elicits a cognitive reappraisal of challenging aspects of the course as opportunities to grow and learn (Jamieson et al., 2022; Rozek et al., 2019). For example, one student wrote, “Lab is fun, it can seem like a drag because lab is always a drag. But I have also never learned so many skills in one class.”

Indeed, a common theme was that the labs were fun, for example, “Labs and lab assignments are not only fun and interesting but can be a great way to connect content to new topics and gain important skills you will use later in other biology courses. Overall, I would recommend you work hard in this class while also having fun and choosing what you like to learn about. There are a lot of times in this course where you have the choice to focus on what you are interested in. This is extremely valuable and can make the course more tailored to your interests, so take advantage of it!” Another wrote, “The labs are a very fun way to engage with the content that you learn in lecture, so make sure to ask questions and understand why you are doing each step in the procedures!” And another, “Get ready for a fun and exciting class and lab, where you will learn so much about the field of neuroendocrinology…” Other students commented on how informative the labs were and the connections between what they were learning in the classroom: “The lab is super informative and will teach you so many things about different hormonal processes in a hands-on way.”

## DISCUSSION

This lab project led to extremely high student motivation and provided students with hands-on experiences using foundational neuroendocrinological methods—as both the researcher and the subject. The students learned about saliva sampling, human subject procedures, and the use of enzyme-linked immunosorbent assays by completing the steps via a simulated ELISA. They carried out the analysis of their own data from raw optical density data to conducting statistical analyses, generating graphs, and interpreting the results. As a part of the process, students read and presented on an original study investigating salivary cortisol responses to psychosocial stressors, which helped to solidify their understanding of the activities. They also put all these pieces together in the form of a substantial deliverable, which required them to synthesize these components and communicate their findings to a general audience.

Through this lab experience, the students discovered that giving a graded, in-class presentation which included questions from their peers, led to a significant increase in salivary cortisol in students. This is consistent with previous studies that found a cortisol increase following assessments in real-life academic settings (Merz & Wolf, 2015; Preuß et al., 2010). Previous research has reported that cortisol responses in the TSS test are blunted in oral contraceptive users (Gervasio et al., 2022). We did not ask about oral contraceptive use here, because of the risk of private medical information becoming known to other students in the course. However, Merz and Wolf (2015) found that a graded oral presentation led to a consistent increase in cortisol in men, women taking hormonal contraceptives, and free-cycling women. I have now conducted a version of this experiment in three different courses, and each time have found this increase in cortisol to be robust, despite the high likelihood of oral contraceptive use among students (72% of the students in the course identified as female).

The simulated ELISA, for which we developed a modified protocol from the kit available via Carolina Biologicals, can be adapted to suit many different contexts. One particularly valuable learning outcome is understanding the use of the standard curve for quantification of a substance, where an unknown sample is compared to a series of diluted controls of known concentrations. Notably, it is also possible to scale down and carry out the protocol in a classroom without pipettors and use the kit’s transfer pipettes (one drop of liquid is equal to approximately 50µL). Thus, this simulated ELISA can be adapted for use even in classes without labs. One note, however, is that there was occasional confusion between the protocol used for the simulated ELISA and the ‘real’ ELISA used to generate the data when students wrote the methods for their poster.

The portion of the lab in which the students developed the survey required them to integrate what they learned about stress in the course, personal experience, and study design principles to design an effective survey. Although only one measure—the students’ self-reported stress with public speaking—was found to be significant in this study, a greater number of measures have been found to be significantly associated with cortisol responses in other iterations of this CURE. Notably this survey is the most student-driven, inquiry-based aspect of the course and, as such, it required appropriate scaffolding and class time to implement successfully.

Additionally, there was substantial variation in students’ previous exposure to statistics prior to this course, which can necessitate additional scaffolding for students to understand the analyses and interpretation of the data. In some scenarios, students will have completed a methods and statistics course prior to upper-level laboratory classes. In our case, however, statistics was not a prerequisite for the course, so we could not assume that the students had any statistical background. As a result, I did two things. First, I had a separate lab that was focused on general data analysis using data from HormoneBase (Vitousek et al., 2018), which had students carry out both t-tests and linear regressions based on their hypotheses in groups. Second, I devoted an entire lab period to walking students through the data curation, quality control, and data analysis of the cortisol and survey data to ensure that the learning outcomes were achieved. However, this aspect of the course would be undoubtedly easier if it was not students’ first exposure to these statistical techniques.

Of course, this project is not without challenges. First, it entails a great deal of work for the instructor and support staff. For example, I wrote protocols and obtained approval of the biosafety committee to carry out the work, in addition to IRB approval to publish this study. There is substantial set up time required to prepare for the labs, including acquiring materials and preparing for each lab. Additionally, this study requires the time and expertise to process the samples, though note that it is possible to send the samples out and have a company process the samples for a fee (e.g. https://salimetrics.com/analyte/salivary-cortisol/). Most importantly, costs may be prohibitive in certain contexts. A BSL-2 lab space and access to key equipment (centrifuge, plate reader, orbital shaker) are all required for completing the ELISA in-house. The kits and supplies here are somewhat expensive, though cheaper options exist through other companies.

This lab exercise can be implemented in both biology or psychology courses with students from diverse backgrounds. The lab activities may also be modified to use any number of other independent variables including exercise, athletic competition, circadian rhythms, or other student stressors, such as sleep deprivation or oral/written exams. Allowing students to choose the independent variable would bring this lab exercise closer to a true course-based research experience (CURE) by increasing the inquiry-based elements, broader potential applications, and increased iteration (Auchincloss et al., 2014). Overall, the results shown here suggest promise for course-based authentic research experiences like this to increase student desire and preparedness to continue in neuroendocrinology, biology, neuroscience and STEM more broadly.

### Address correspondence to:

Dr. Nicole M. Baran, Department of Biology, Davidson College, 209 Ridge Rd, Box 5000, Davidson, NC 28036. Email: nmbaran@davidson.edu

## Supplementary Material

ELISA Simulation Prep

Student Designed Survey

Simulated ELISA Protocol & Worksheet

Salivary Cortisol Collection Procedure
