# Supplementary material for: An Inquiry-Based Lab Exercise Investigating Student Cortisol Responses to Giving a Graded In-Class Presentation
Source: J Undergrad Neurosci Educ. 2026 Apr 23;24(2):17–24. doi: 10.59390/001c.157973 (PMC13625904; doi:10.59390/001c.157973)
Supplement: Supplementary file 1 [file junejournal_2026_24_2_157973_331905.docx]

**ELISA Simulation Preparation Protocol**

**Serial Dilution of Positive Control for Standard Curve**

The below chart is for the stock solutions, then they will need to be aliquoted.

Requires two kits worth of reagents.

| Labels on 1.5ml microcentrifuge tubes | Amount from  previous soln | Water Amount to be added | Aliquot totals x 16 |
| --- | --- | --- | --- |
| 3.0 std | Positive Control Tube | Nothing added | 200 µl |
| 1.0 std | 5 mls previous tube | 5 ml H_2_0 | 200 µl |
| 0.333 std | 5 mls previous tube | 5 ml H_2_0 | 200 µl |
| 0.111 std | 5 mls previous tube | 5 ml H_2_0 | 200 µl |
| 0.037 std | 5 mls previous tube | 5 ml H_2_0 | 200 µl |
| 0.012 std | 5 mls previous tube | 5 ml H_2_0 | 200 µl |
|  |  |  |  |
| Positive Control | Nothing added | Nothing added | 200 µl |
| Negative Control | Nothing added | Nothing added | 200 µl |

**Sample Dilutions and Labels**

| Label on 1.5 ml microcentrifuge tubes | Actual sample from kit | Dilution Factor  (with H_2_0) | Aliquot totals x 16 |
| --- | --- | --- | --- |
| A1 | A | 1/3 | 200 µl |
| A2 | A | 1/2 | 200 µl |
| B1 | A | 1/2 | 200 µl |
| B2 | A | 1 | 200 µl |
| C1 | D | 1/2 | 200 µl |
| C2 | D | 1 | 200 µl |
| D1 | D | 1 | 200 µl |
| D2 | D | 1 | 200 µl |
| E1 | F | 1/4 | 200 µl |
| E2 | F | 1/3 | 200 µl |
| F1 | F | 1/2 | 200 µl |
| F2 | F | 1 | 200 µl |

| Label on 10 or 15 ml conical tubes | Actual Name from Kit | Aliquot totals x 16 |
| --- | --- | --- |
| Assay Diluent | Antigen | 3.5 mls |
| Enzyme Conjugate | Secondary Antibody | 3.5 mls |
| TMB Substrate | Chromogen | 3.5 mls |

**Additional Supplies Needed for Students**

- Microtiter plates x 16
- Versi dry lab paper
- P200 Pipettes
- Regular pipet tips

Plates can be run on a plate reader to compute standard curve R^2^ at 400 nm
