# Supplementary material for: An Inquiry-Based Lab Exercise Investigating Student Cortisol Responses to Giving a Graded In-Class Presentation
Source: J Undergrad Neurosci Educ. 2026 Apr 23;24(2):17–24. doi: 10.59390/001c.157973 (PMC13625904; doi:10.59390/001c.157973)
Supplement: Supplementary file 2 [file junejournal_2026_24_2_157973_331906.docx]

**Student-designed survey**

**Questions 1-7 are student-designed questions, as discussed in class:**

1. How many hours do you sleep each school night, on average?
2. How many hours do you spend on all of your schoolwork (outside of class) per week?
3. How many hours do you spend each week on extracurricular activities, clubs, athletics, etc…?
4. How many hours do you spend working at a paid job per week? (write zero (0) if you do not currently have a job)
5. How would you rate your overall level of stress in the last month? (using the scale of 1 to 5, where 1 is the lowest and 5 the highest)?

*Rate the following statements using the following scale (1=Strongly Disagree, 2=Disagree, 3=Neither agree nor disagree, 4=Agree, 5=Strongly Agree):*

6. I get the emotional support I need from my family and friends.
7. I find public speaking stressful.

**Questions 8-13 are from the six-item short form six-item short-form of the state scale of the Spielberger State-Trait Anxiety Inventory (STAI) (Marteau & Bekker, 1992; Spielberger, 1982) and is a widely used and validated measures of anxiety.**

Rate the following statements using the following scale: 1 = Not at all, 2 = Somewhat, 3 = Moderately so, 4 = Very much so

8. I feel calm.
9. I feel tense.
10. I feel upset.
11. I feel relaxed.
12. I feel content.
13. I feel worried.

**References**

Marteau, T. M., & Bekker, H. (1992). The development of a six-item short-form of the state scale of the Spielberger State—Trait Anxiety Inventory (STAI). *British Journal of Clinical Psychology*, *31*(3), 301–306. <https://doi.org/10.1111/j.2044-8260.1992.tb00997.x>

Spielberger, C. D., Gorsuch, R. L., Lushene, R., Vagg, P. R., & Jacobs, G. A. (1983). Manual for the state-trait anxiety scale. *Consulting Psychologists*.
