# Supplementary material for: An Inquiry-Based Lab Exercise Investigating Student Cortisol Responses to Giving a Graded In-Class Presentation
Source: J Undergrad Neurosci Educ. 2026 Apr 23;24(2):17–24. doi: 10.59390/001c.157973 (PMC13625904; doi:10.59390/001c.157973)
Supplement: Supplementary file 3 [file junejournal_2026_24_2_157973_331907.docx]

**Laboratory exercise:**

**Simulated Enzyme-Linked Immunosorbent Assay**

**Introduction**

This simulated Enzyme-Linked Immunosorbent Assay (ELISA) from Carolina Biologicals (<https://www.carolina.com/gene-expression-advanced-topics/elisa-simulation-kit/FAM_211248.pr>) has been adapted to simulate a direct competitive immunoassay (see figure below). In the real ELISA kit from Salimetrics (<https://salimetrics.com/assay-kit/salivary-cortisol-elisa-kit/>), cortisol in standards and samples compete with cortisol conjugated to horseradish peroxidase for the antibody binding sites on a microtitre plate. After incubation, unbound components are washed away. Bound cortisol enzyme conjugate is measured by the reaction of the horseradish peroxidase enzyme to the substrate tetramethylbenzidine (TMB). In the real ELISA, this reaction produces a blue color. A yellow color is formed after stopping the reaction with an acidic solution. The optical density/aborption is read on a standard plate reader at 450 nm. The amount of cortisol enzyme conjugate detected via the color reaction is inversely proportional to the amount of cortisol present in the sample.

In this simulated ELISA, all reaction wells will turn light green when the TMB substrate solution is added and then a color change from light green to purple indicates the presence of cortisol. The depth of the color is correlated with the concentration of cortisol in the sample.

Source: <https://www.mblbio.com/bio/g/support/method/elisa.html>

**Question: In the real ELISA, is a darker yellow color associated with more cortisol or less cortisol in the sample? Explain.**

**Instructions**

**Supplies needed:**

- Microtitre plate
- Cortisol standards (3.0, 1.0, 0.333, 0.111, 0.037, 0.012 µg/dL and H_2_O)
- Cortisol controls (High & Low)
- Precision pipettors (p200s)
- Pipette tips
- Assay diluent
- Enzyme conjugate (i.e. enzyme labeled antigen)
- TMB Substrate solution

**Protocol:**

1. Using a p200 pipette, add 75µL of each of your **cortisol standards** *in duplicate* (columns 1-2) to rows A-F. Use a fresh pipette tip for each of the standards.
2. Add 75µL of each of your **positive (+) and negative (-) controls** to the appropriate wells (columns 1-2, rows G & H)
3. Add 75µL of each of your **samples** *in duplicate* in columns 3-4 (rows A-H) and columns 5-6 (rows A-D). Use a fresh pipette tip for each sample. See below.
4. Add 75µL of **assay diluent** to each well. You do not need to change tips, as long as you are careful to not touch the liquid inside the wells.
5. Add 75µL of **enzyme conjugate** to each well. You do not need to change tips, as long as you are careful to not touch the liquid inside the wells. You do not need to worry about mixing your samples here. You may gently swirl the plate on the bench, but in a real ELISA, we would use a plate rotator to gently mix the solutions in the wells of the microtitre plate.
6. NOTE: If this was a real ELISA, there would be a series of wash steps at this point to remove all of the unbound molecules. This step has been eliminated from the simulation.
7. Add 75µL of **TMB substrate**. All the wells will turn light green when the TMB substrate is added. A change from light green to purple indicates the presence of cortisol in the sample. Incubate the microtitre plate at room temperature for 5-10minutes to allow for color development.
8. After 5-10 minutes of incubation, we will read the colors on a plate reader at 4000 nm to obtain a highly precise measure of absorbance for each well. In addition, take a picture of your plate, ideally with a white background!

|  | **1** | **2** | **3** | **4** | **5** | **6** |
| --- | --- | --- | --- | --- | --- | --- |
| **A** | 3.0 std | 3.0 std | A1 | A1 | E1 | E1 |
| **B** | 1.0 std | 1.0 std | A2 | A2 | E2 | E2 |
| **C** | 0.33 std | 0.33 std | B1 | B1 | F1 | F1 |
| **D** | 0.11 std | 0.11 std | B2 | B2 | F2 | F2 |
| **E** | 0.037 std | 0.037 std | C1 | C1 |  |  |
| **F** | 0.012 std | 0.012 std | C2 | C2 |  |  |
| **G** | Neg | Neg | D1 | D1 |  |  |
| **H** | Pos | Pos | D2 | D2 |  |  |

**Best Practices:**

- Always wear gloves.
- When in doubt, change tips.
- Use a system for keeping track of what you have added.

**Additional Question**

**How does the standard curve enable you to calculate the concentration of an antigen (in this case cortisol) in a sample? Briefly explain the steps.**

**Does each individual microtiter plate need its own standard curve? Why?**

**Why are samples run in duplicate or triplicate? What should you do if the two samples are very different from each other?**

**On the real plate, there are a series of controls. What do each of the controls tell you?**

**Zero/negative control:**

**Non-specific binding (NSB, does not contain anti-cortisol antibodies):**

**High cortisol control:**

**Low cortisol control:**
