# Supplementary material for: An Inquiry-Based Lab Exercise Investigating Student Cortisol Responses to Giving a Graded In-Class Presentation
Source: J Undergrad Neurosci Educ. 2026 Apr 23;24(2):17–24. doi: 10.59390/001c.157973 (PMC13625904; doi:10.59390/001c.157973)
Supplement: Supplementary file 4 [file junejournal_2026_24_2_157973_331908.docx]

**Laboratory Exercise:**

***Salivary Cortisol Sample Collection Procedure***

Cortisol (CORT) is a steroid hormone that is released in response to stress, and which can be detected non-invasively in saliva samples. For this lab activity, we will conduct a simple experiment looking at salivary cortisol concentrations in students in the course at baseline during a normal lab day and following a brief psychosocial stressor (giving a presentation on a scientific journal article for a grade). This is a slight modification of a protocol known as the Trier Social Stress Test, which reliably induces CORT elevations in laboratory settings.

**Biosafety information & precautions:** Biosafety measures are in place and all the sample collection and handling procedures have been approved by Davidson’s Institutional Biosafety Committee (IBC), in accordance with federal regulations. Saliva is ordinarily considered a non-hazardous body fluid unless visibly contaminated with blood. However, the COVID-19 pandemic has greatly changed the risk profile for collection and handling of saliva. Recent studies have found that the saliva of infected persons had high levels of virus and were comparable to standard nasal swabs in sensitivity and specificity. Therefore, saliva must be considered a high-risk source of viral exposures, and *the collection and handling of saliva must implement biosafety procedures to mitigate the risk of exposure*.

Therefore, you are responsible for following all instructions to ensure the safety of yourself and your peers. Please read the instructions below for sample collection and follow the procedures carefully. *No students will interact with samples other than their own*. Once samples are collected, all work with samples will be carried out by the instructor in lab space that has been certified as a modified [Biosafety Level 2](https://www.phe.gov/s3/BioriskManagement/biosafety/Pages/Biosafety-Levels.aspx), which provides protections to enable the study of moderate-risk infectious agents. We will use a commercially available enzyme-linked immunosorbent assay (ELISA) kit ([Salimetrics Assay #1-3002](https://salimetrics.com/assay-kit/salivary-cortisol-elisa-kit/)) to measure cortisol concentrations in the saliva samples. Samples will be analyzed in duplicate. We will then provide the class with the raw data, and you will be responsible for statistically analyzing the data, creating figures, and creating a scientific poster based on this study.

___________________________________________________________________________

**Before you collect the sample:**

It is **required** that you wait at least 30 minutes after eating, taking medicine, or brushing your teeth before collecting a sample. You may find it helpful to drink water between samples – this should not affect hormone concentrations as long as you swallow the water before putting the sponge in your mouth.

Your unique identifier for this lab is on page 3. Please write this code on each of your samples and use it during surveys so that we can connect your samples together. **Take a picture or write the code somewhere in your notes where you’ll be able to find it**. To protect everyone’s confidentiality, do NOT share your identifier with anyone else in the class.

**Please follow all instructions completely!**

**Taking a sample:**

1. Wash your hands with soap and hot water using the sink.
2. Use a Lysol wipe to wipe down the surfaces in the collection area and place the Lysol wipe in the zip-lock bag labelled “waste”.
3. Rinse your mouth with water (you can swallow this).
4. Place a absorbent plastic-backed sheet (soft side up) on the surface over which you'll be doing your collection.

5. Remove the cap of the tube so you can see the sponge. Do not remove the holder the sponge is inserted into.
6. Tip the sponge into your mouth from the tube without touching it.
7. Gently chew and roll the sponge in your mouth for about 1 minute until it's saturated. Then use your teeth and tongue to gently place the sponge back in the tube without touching the sponge and avoiding generating aerosols.
8. Put the cap back on the tube containing the sponge. Be certain the tube is tightly closed!
9. If there is any evidence of blood in the sample, please double bag your sample and place the sample into the waste bag. You should then wash your hands and notify the instructor so that the sample can be disposed of properly.
10. Disinfect hands and collection tube with an alcohol-based sanitizing wipe.
11. Using the available sharpie marker, label the tube with the date, time, sample number [1 (baseline) *OR* 2 (treatment)], and your unique three-digit code (in the name row). (Please do NOT include your birth date!)
12. Place the tube in the plastic zip-lock bag labeled "completed sample".
13. Wipe your face and hands with a paper towel and place them in the zip-lock bag labelled "waste." Fold up the blue sheet so that only the plastic side is showing, and also place it in the zip-lock waste bag.
14. Use a Lysol wipe to wipe down the surfaces in the collection area and place the Lysol wipe in the waste bag. Seal the waste bag.
15. Finally, wash your hands with soap and hot water using the sink. This paper towel may go into the regular trash.
16. It is critical that you maintain control of your samples. After you’ve collected your samples, gather your things, and bring your sample AND waste bag directly back to the classroom and hand them to the instructor.
17. Use one of the sinks to quietly wash your hands again with soap and hot water and then return to your seat.

Your unique identifier for this lab is _______. Please write this code on each of your samples and use it during surveys so that we can connect your samples together. **Take a picture or write the code somewhere in your notes where you’ll be able to find it**. To protect everyone’s confidentiality, do NOT share your identifier with anyone else in the class.

**Symptom Questionnaire**

- Do you have a **fever** (temperature over 100.4º F or 38º C) without having taken any fever-reducing medications?
- Do you have a **loss of smell or taste**?
- Do you have a **cough**?
- Do you have **muscle aches**?
- Do you have a **sore throat**?
- Do you have **shortness of breath**?
- Do you have **chills**?
- Do you have a **new or unusual headache**?
- Have you experienced new onset of any **gastrointestinal symptoms** such as nausea, vomiting, diarrhea, or loss of appetite in the last few days?
- Have you **tested positive for COVID-19** in the past 10 days?
- Are you **currently awaiting results** from a COVID-19 test?
- Have you been **diagnosed with COVID-19** by a licensed healthcare provider (for example, a doctor, nurse, pharmacist, or other) in the past 10 days?

If you answer yes to ANY of these questions, please do NOT provide a sample.

There will be no impact to your experience in the course!
